# SAGIT^®^: clinician-reported outcome instrument for managing acromegaly in clinical practice—development and results from a pilot study

**DOI:** 10.1007/s11102-015-0681-2

**Published:** 2015-09-16

**Authors:** Andrea Giustina, John S. Bevan, Marcello D. Bronstein, Felipe F. Casanueva, Philippe Chanson, Stephan Petersenn, Xuan-Mai Truong Thanh, Caroline Sert, Aude Houchard, Isabelle Guillemin, Shlomo Melmed, The SAGIT Investigator Group

**Affiliations:** Chair of Endocrinology, University of Brescia, Brescia, Italy; Aberdeen Royal Infirmary, Aberdeen, UK; Hospital das Clínicas da Universidade de São Paulo, São Paulo, Brazil; Department of Medicine, Santiago de Compostela University - CIBER de Fisiopatología de la Obesidad y Nutrición (CIBERobn), Instituto Salud Carlos III, Santiago de Compostela, Spain; Hôpital Bicêtre, Kremlin-Bicêtre, France; ENDOC Centre for Endocrine Tumours, Hamburg, Germany; Ipsen, Boulogne-Billancourt Cedex, France; Mapi, Lyon, France; Cedars-Sinai Medical Center, Los Angeles, CA USA

**Keywords:** Acromegaly, Clinician-reported outcomes, Instrument, Pilot study

## Abstract

**Purpose:**

The SAGIT instrument is a comprehensive clinician-reported outcome instrument assessing key features of acromegaly: signs and symptoms, associated comorbidities; growth hormone levels; insulin-like growth factor-1 levels; and tumor profile. The SAGIT instrument has been designed to assist endocrinologists managing acromegaly in practice. Here, we report on pre-testing (to assess ease of understanding and acceptability) and a pilot study (to assess relevance, ease of use, and utility in real-life conditions) (NCT02231593).

**Methods:**

For pre-testing, 11 endocrinologists completed the SAGIT instrument using patient medical records and were also interviewed. They subsequently completed a PRAgmatic Content and face validity Test (PRAC-Test^©^) to report their experiences using SAGIT, and feedback was used to revise the instrument. In the pilot study, nine endocrinologists completed the SAGIT instrument in real-time with patients belonging to three different categories (stable/controlled, active/uncontrolled acromegaly, treatment-naïve), while four completed the instrument based on medical-record review. All participants then completed the PRAC-Test^©^ and their feedback was used to update the instrument.

**Results:**

The SAGIT instrument was well accepted by endocrinologists, with most indicating that it was concise, practical, easy to understand, useful for assessing treatment response, and valuable as a component of the patient’s medical record. The pilot study confirmed the instrument’s acceptability, utility, and ease of use, and indicated its potential for distinguishing acromegaly clinical stages.

**Conclusions:**

The SAGIT instrument is promising as a tool for use by endocrinologists in everyday practice to assess the status and evolution of disease in patients with acromegaly and to guide treatment decision-making.

**Electronic supplementary material:**

The online version of this article (doi:10.1007/s11102-015-0681-2) contains supplementary material, which is available to authorized users.

## Introduction

Acromegaly is a chronic multisystem disease resulting from the oversecretion of growth hormone (GH), which is usually caused by a pituitary adenoma [1, 2]. This leads to an overproduction of insulin-like growth factor-1 (IGF-1), with consequent somatic overgrowth and physical disfigurement [3, 4]. Acromegaly can cause a variety of symptoms, such as sweating, headache, and joint pain [5], and is associated with severe comorbidities [3]. Diagnosis is based on assessment of biochemical (GH and IGF-1) and imaging (pituitary tumor) components, as well as clinical features [3, 6]. The main treatment options are surgery and long-acting somatostatin analogs in those who cannot be cured with surgery or who are poor surgical candidates [3, 7, 8]. The goals of treatment are to ameliorate symptoms, reduce morbidity and mortality, and control GH/IGF-1 hypersecretion and tumor growth [3, 8, 9]. However, clinical features and the biochemical profiles may give discordant information, which hampers diagnostic and decision-making processes [3]. In addition, there may be discrepancies between the results of GH and IGF-1 assays [10, 11], further emphasizing the need for comprehensive and integrative evaluation of all disease-specific parameters.

**Fig. 1:**
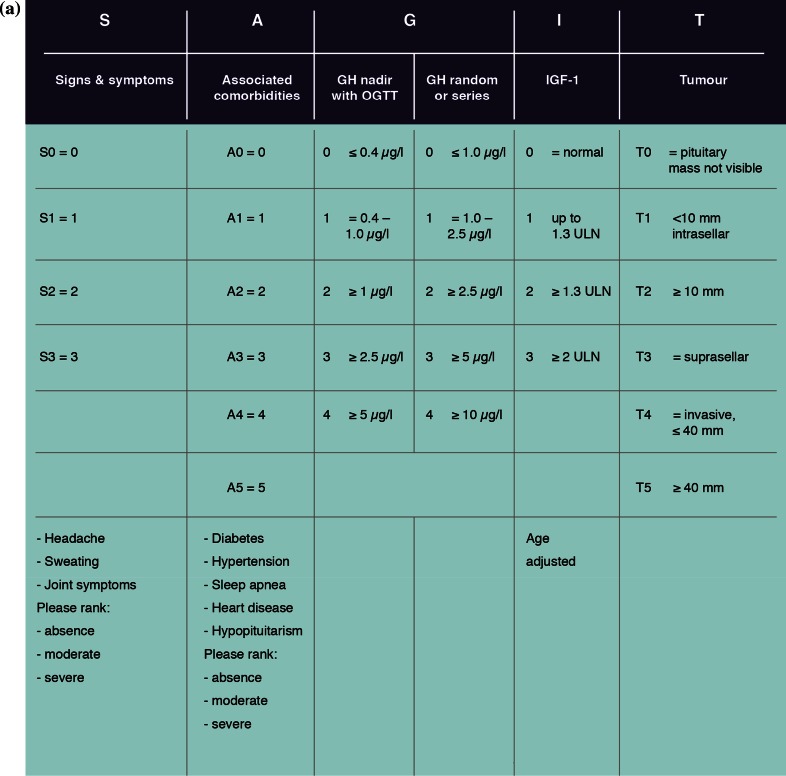

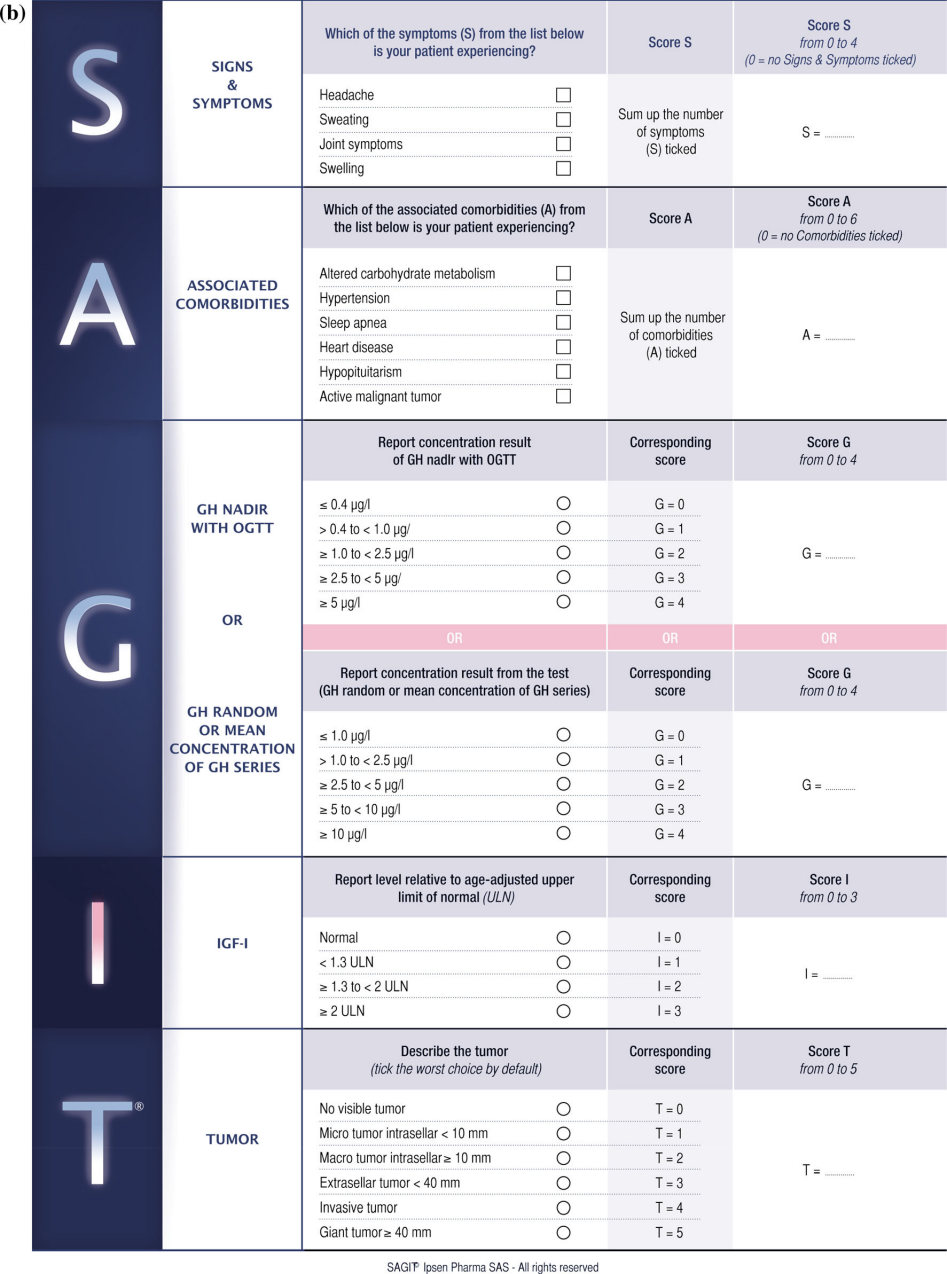
Versions of the SAGIT instrument **a** used in Step 1 pre-testing (original version) and **b** following completion of Step 1 pre-testing and Step 2 pilot study

Two patient-reported outcome (PRO) instruments are currently available for assessing acromegaly: the Patient-assessed Acromegaly Symptom Questionnaire (PASQ) [12] and the Acromegaly Quality of Life (AcroQoL) questionnaire [13]. However, neither record the full spectrum of features (biochemical, tumoral, and clinical) necessary to optimally diagnose, stage and manage acromegaly. In addition, a previous study has shown that the structure and function of pituitary adenomas may be useful for classifying acromegaly types [14]. Thus, there is a need for a comprehensive instrument that records biochemical, tumoral, and clinical aspects of acromegaly. Two such instruments are currently in development: SAGIT and ACRODAT (ACROmegaly Disease Activity Tool) [15]. The SAGIT instrument is multidimensional, comprising five sections that assess key features of acromegaly [6]: signs and symptoms (S), associated comorbidities (A), GH levels (G), IGF-1 levels (I), and the Tumor profile (T) (Fig. 1a).

The SAGIT instrument was developed by a steering committee comprising acromegaly experts from the Acromegaly Consensus Group, which has published guidelines and consensus papers [16–20]). The aim was to provide a reference instrument for acromegaly staging in clinical practice. A global development program, involving an iterative, 3-step process (NCT02231593), was implemented to develop and validate the original SAGIT instrument. Step 1 pre-testing evaluated ease of understanding and acceptability of the instrument and its layout by future users (i.e. endocrinologists). In the Step 2 pilot study, endocrinologists assessed the interface and also content validity, relevance, and acceptability. The Step 3 clinical validation phase will consist of a large clinical study to evaluate performance of the instrument, develop scoring, determine score thresholds to stage patients and assess treatment response, and develop decision indications for patient management. Here, we report the methodology and results from the pre-testing and pilot study steps.

## Methods

The process for development and validation of the SAGIT instrument (Supplemental Figure 1) was based on the methodology of Arnould [21]. Members of the steering committee were involved at all key milestones of the project; after each step, a steering-committee meeting was held to discuss the results and agree subsequent steps.

### Step 1 pre-testing

The objectives of Step 1 pre-testing were: to assess practising endocrinologists’ understanding of the original version of the SAGIT instrument and to assess its layout and length; to explore understanding and acceptability in different countries; and to evaluate its relevance, ease of use, applicability, and usefulness in practice.

Testing was conducted in Brazil, France, Germany, Italy, Spain, and the UK using retrospective evaluation of patients’ medical records. Twelve endocrinologists (two per country) were asked to participate, each assessing de-identified medical records of three patients. Endocrinologists were eligible for inclusion if they were currently treating at least three patients with acromegaly and were not familiar with the SAGIT instrument. Medical records were included if patients were aged ≥18 years, had a confirmed diagnosis of acromegaly, and had given consent for the use of their (acromegaly) medical information.

Each endocrinologist was required to complete the SAGIT instrument on two occasions for each patient [before and ≥3 months after an intervention (i.e. medical treatment, surgery, or radiotherapy)]. Endocrinologists also completed the evaluation form for the PRAgmatic Content and face validity Test (PRAC-Test^©^) and they participated in a 1-h cognitive debriefing interview (conducted by telephone) to assess their understanding and opinion of the items in each section of the SAGIT instrument. The PRAC-Test^©^ is a standardized, rigorously developed questionnaire designed to systematically evaluate the qualities and limitations of patient-reported outcomes instruments in clinical practice [22]. It is also applicable to clinician-reported outcome (ClinRO) instruments.

### Step 2 pilot study

Following Step 1 pre-testing, the SAGIT instrument was updated to reflect feedback from the endocrinologists. The resulting SAGIT instrument (pilot version) was then tested in the Step 2 pilot study. The objectives of the pilot study were: to evaluate the updated version of the SAGIT instrument in patients with acromegaly being actively managed in real-time conditions in clinical practice; to evaluate the acceptability of the updated instrument to practising endocrinologists and their intention to use it in clinical practice; and to finalize an operational version of the SAGIT instrument for use in the clinical validation study.

The pilot study used a prospective, multicenter, observational design in five countries (France, Germany, Italy, Spain, and the USA), where ethics approval was obtained (Step 2 prospective pilot study). Endocrinologists in these countries completed a copy of the SAGIT instrument for each patient, as well as a copy of the PRAC-Test^©^ evaluation form. Due to time constraints with ethics procedures in the UK and Brazil, the retrospective design from Step 1 (i.e. using patient records combined with a cognitive debriefing interview) was used in these countries (Step 2 retrospective pilot study).

Fourteen endocrinologists (two per country) participated, and each was to assess three patients (or the medical records of three patients): one treated patient with stable/controlled acromegaly, one treated patient with active/uncontrolled acromegaly, and one treatment-naïve patient. Inclusion criteria for the endocrinologists were the same as Step 1; as familiarity with the SAGIT instrument was an exclusion criterion, those who participated in Step 1 were not eligible to take part in Step 2. Patient selection criteria were the same as those for Step 1; these and specific inclusion criteria for stable/controlled, active/uncontrolled, and treatment-naïve subgroups are summarized in Table 1.

**Table 1:** Patient inclusion criteria for the Step 2 pilot study

All patients:
• Male or female, ≥18 years of age • Diagnosis of acromegaly confirmed by IGF-1 levels >1.3 times the ULN, GH levels >0.4 µg/L after an oral glucose load (75 g), and presence of a pituitary adenoma on MRI (note that the diagnosis had to be suspected by a physician prior to testing and not an incidental finding during work-up of non-pituitary or endocrine complaint) • Cognitive and linguistic capacity to understand information provided on the conditions and objectives of the study
Controlled/stable patients:
• Patients who had received medical treatment or surgery ≥3 months previously • Those with an MRI scan in the previous 12 months that reflected the current treatment phase (i.e. post-surgical or post-medical therapy) and corresponded to the patient’s current disease status • Those with stable, controlled biochemical disease, demonstrated by: • ≥2 IGF-1 measurements within the normal range (both samples had to be taken prior to entry, ≥1 month apart, and during treatment with the same medication regimen or post-surgery without medication) • GH suppression to <0.4 µg/L following an oral glucose tolerance test (in patients who were post-surgical and not requiring medication) or GH levels < 1 µg/L (in those treated with somatostatin receptor ligands); patients treated with pegvisomant were not required to demonstrate control by measurement of GH • **No** history of non-compliance or inability to reliably receive treatment in the foreseeable future • **No** gaps in treatment of > 1 month within the 12 months prior to study entry
Patients with active/uncontrolled acromegaly:
• Patients who had received medical treatment or surgery ≥3 months previously • Those with an MRI scan in the previous 3 months that reflected the patient’s current disease status • Those with stable, controlled biochemical disease, demonstrated by: • ≥2 IGF-1 measurements >1.3 × ULN above the normal range on both occasions (both samples had to be taken prior to entry, ≥1 month apart, and reflect the effect of the patient’s current treatment regimen [or a lower dose of the same medication] or the effect of the disease prior to treatment initiation) • GH levels >1 µg/L after surgery or before medication administration if surgery is not performed
Treatment-naïve patients:
• Patients who had not received any form of treatment prior to study entry, including surgery, medication, or radiation.

*GH* growth hormone, *IGF-1* insulin-like growth factor-1, *MRI* magnetic resonance imaging, *ULN* upper limit of normal

## Results

### Step 1 pre-testing

#### Characteristics of participating clinicians

Twelve endocrinologists were recruited; of these, 11 completed the SAGIT instrument (based on the medical records of 33 patients) and the PRAC-Test^©^ questionnaire, and 10 were interviewed. Participating endocrinologists were aged 32–58 years, with most working in a hospital environment (Table 2). The number of acromegaly patients seen by the endocrinologists varied across participating countries, from 2–5 per month in the UK to 10–60 per month in Brazil (Supplemental Table 1).

**Table 2:** Characteristics of the endocrinologist population included in Step 1 pre-testing and Step 2 pilot study

Characteristics	Step-1 pre-testing (n = 11)	Step 2 pilot study	
		Prospective (n = 9)	Retrospective (n = 4)
Age (years)^a^	32–58	34–56	38–48^b^
Mode of practice			
Outpatient clinic	4	3	4
Hospital	9	8	0
Number of years treating acromegaly patients^a^	2–30	3–30	10–19
Number of acromegaly patients seen per month^a^	2–60	3–15	1–80

Some endocrinologists worked in both outpatient clinics and hospitals. A breakdown of data by country are shown in Supplemental Table 1

^a^Range

^b^Missing data for one endocrinologist

#### Telephone interview

Endocrinologists indicated that the SAGIT instrument was easy to use and understand and that it was simple to complete. It was considered useful for clinical practice, providing a standardized approach for assessing disease progression and the effects of new interventions. Several areas for improvement were identified, including: how to report and interpret the score for each section; how to use the information on severity ranking for the signs and symptoms (S) and associated comorbidities (A); how the scores could be used to guide patient management; and correlation between scores and prognosis/treatment recommendations.

Endocrinologists’ feedback on items in each section of the SAGIT instrument is summarized in Supplemental Table 2. This included: the proposal of additional signs and symptoms and associated comorbidities; the subjectivity of the severity ranking in these sections; and confusion on how to report GH levels (specifically, whether one or both measures were required).

#### *PRAC*-*Test*^*©*^

**Fig. 2:**
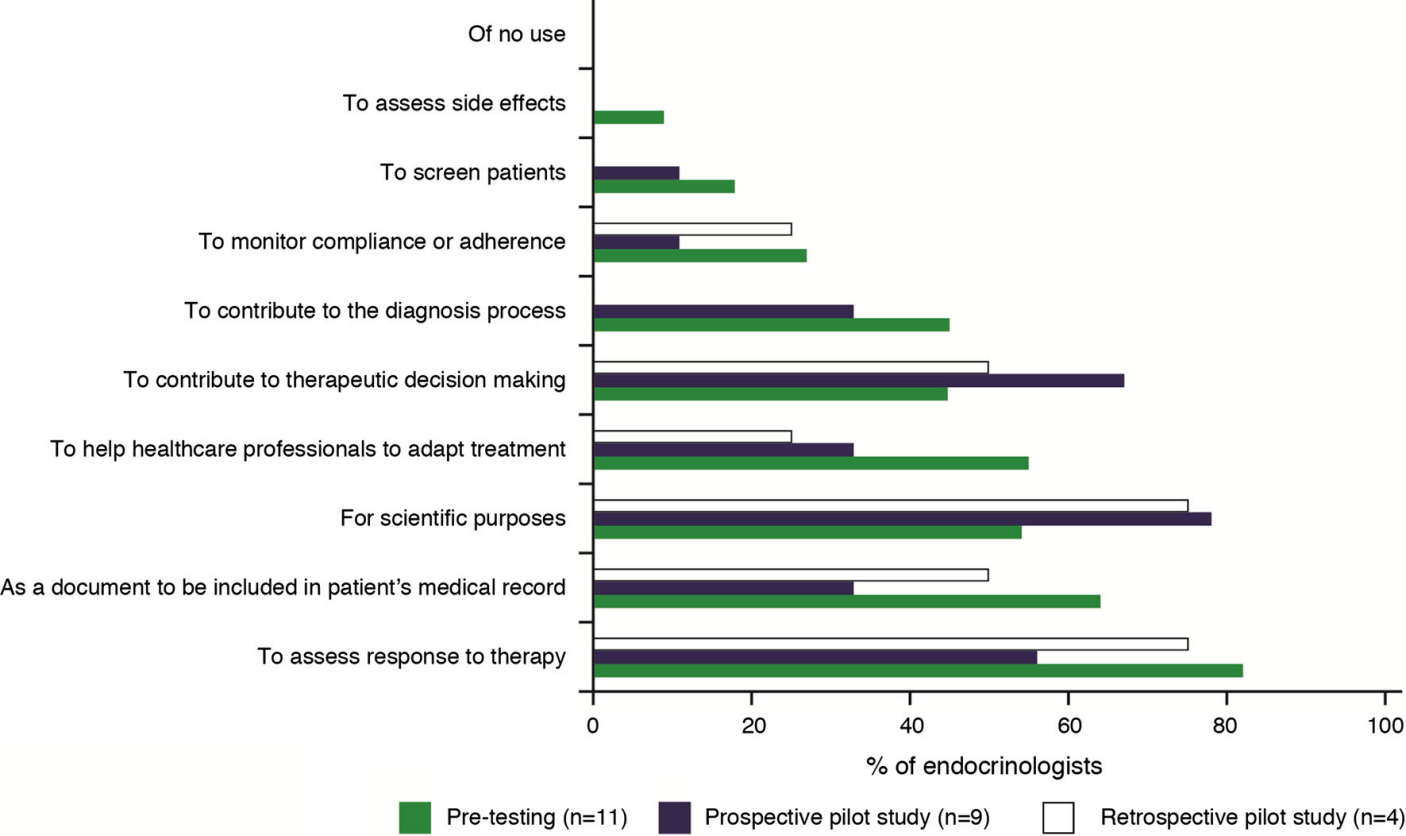
Utility of the SAGIT instrument: results of PRAC-Test^®^ questionnaire during Step 1 pre-testing and Step 2 pilot study. *Note* that multiple responses were possible

Responses to the questions on the utility of the SAGIT instrument are summarized in Fig. 2 and country-specific data are shown in Supplemental Table 3. Most endocrinologists reported that the instrument would be useful for assessing the response to treatment (9/11) and as a component of the medical record (7/11). None of the participants responded that the instrument would be of ‘no use’.

**Fig. 3:**
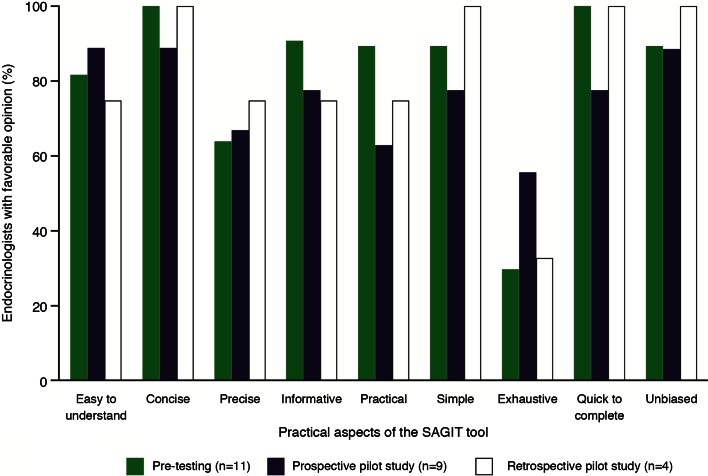
Practical aspects of the SAGIT instrument: results of the PRAC-Test^®^ questionnaire during Step 1 pre-testing and Step 2 pilot study. *Note* that multiple responses were possible

Responses on practical aspects of using the SAGIT instrument are depicted in Fig. 3 (and for the individual countries in Supplemental Table 4). Most endocrinologists indicated that the instrument was concise (11/11), informative (10/11), quick to complete (10/10), easy to understand (9/11), simple (9/10), practical (9/10), unbiased (9/10), and precise (7/11). Most endocrinologists reported that the instrument was not exhaustive (8/10). Four elements were identified as requiring improvement: instructions for completion, the response choices, the scores/decision rules, and the interpretation/recommendations. Most participants felt that the instrument required improvements; the number of participants indicating that *no* further improvements were needed was: title, 5/11; questions, 5/11; interpretation and recommendations, 2/11; instructions, 1/11; response choices, 1/11; score and decision rules, 1/11.

All endocrinologists reported that some parties would benefit from using the SAGIT instrument, including the scientific research community (10/11) and healthcare professionals (6/11). Three participants indicated that patients could benefit and two that the pharmaceutical industry could benefit.

When asked about reasons for not using the SAGIT instrument, 8/11 endocrinologists selected one or more of the following from a list of potential reasons: practicality (1/11), lacks relevance for patients (2/11), lacks clinical relevance (3/11), lacks scientific credibility (2/11), and layout (1/11). Four endocrinologists indicated that there was no reason not to use the instrument. In terms of their willingness to use the SAGIT instrument, one participant indicated that they would not use SAGIT with any of their patients; the remainder would use it with a minority of their patients (2/11), the majority of their patients (5/11), or all of their patients (3/11). Furthermore, nine of the 11 endocrinologists would recommend the instrument to their colleagues and 8/11 believed that they would have a positive reaction to it.

Most participants reported that it would be most appropriate to complete the SAGIT instrument during a patient consultation (7/11), while others noted that it would be best completed between consultations (3/11) or in the waiting room immediately before the consultation (2/11); one participant responded ‘other’.

Following completion of the pre-testing step, the SAGIT instrument was revised to take into account endocrinologists’ feedback. Revisions included addition of swelling to the signs and symptoms section, addition of malignant tumor to the associated comorbidities section, and improved categorization of tumors in the tumor section. Severity rankings were also removed and space was provided to report the score for each section.

### Step 2 pilot study

#### Characteristics of participating clinicians and their patients

Of the 14 endocrinologists recruited, 13 participated in the pilot study, four from Brazil and the UK, and nine from other countries. Overall, 26 patients were included in the prospective pilot study; in the UK and Brazil (retrospective pilot study), the medical records of 12 patients were evaluated. The ages of the participating endocrinologists ranged from 34 to 56 years, with similar numbers working in outpatient clinics and hospitals (Table 2). The number of acromegaly patients encountered by the endocrinologists varied from 1–10 per month in the UK to 12–80 per month in Brazil (Supplemental Table 1).

Of the 26 patients whose data were analyzed in the pilot study, nine had active/uncontrolled acromegaly, 10 had stable/controlled acromegaly, and seven were treatment-naïve. Baseline characteristics of these patients are summarized in Supplemental Table 5.

#### Use of SAGIT instrument to stage disease

**Fig. 4:**
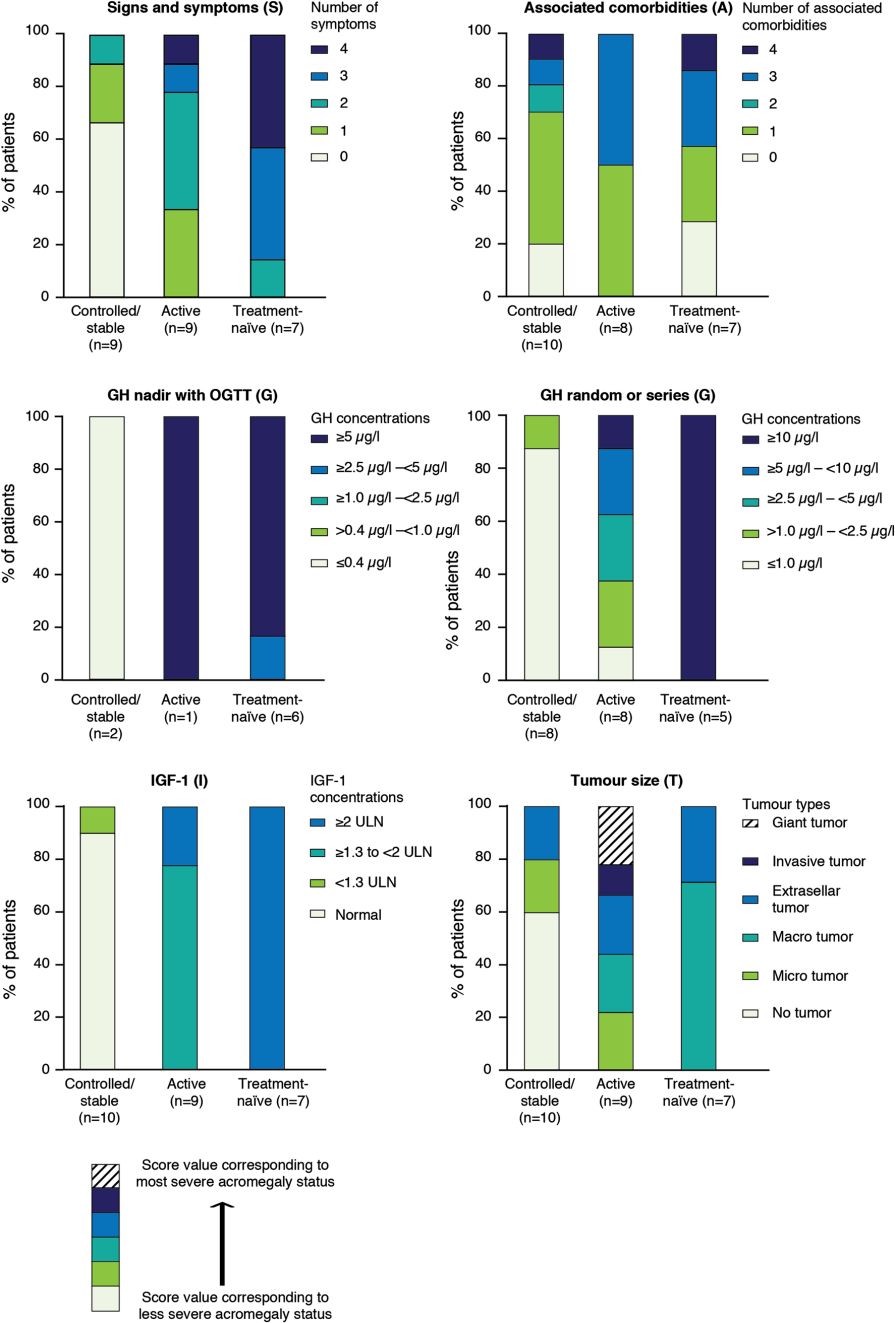
Distribution of endocrinologists’ scores according to patients’ profiles (Step 2 pilot study; n = 26 patients)

The distribution of endocrinologists’ SAGIT scores according to patients’ disease status is summarized in Fig. 4. Most patients with stable/controlled disease were characterized by a lack of signs and symptoms (6/9) and a single comorbidity (5/10), a GH nadir following an oral glucose tolerance test (OGTT) of ≤0.4 µg/L (2/2) or GH random/series ≤1 µg/L (7/8), normal IGF-1 levels (9/10), and no tumor (6/10). For those with active/uncontrolled disease, most had one or two signs and symptoms (7/9), up to three comorbidities (8/8), and IGF-1 levels ≥1.3 × ULN (7/9). GH levels (random/series) varied between 1 and 10 µg/L, and distribution was scattered across the tumor types. Most treatment-naïve patients had three or four signs and symptoms (6/7) and macro-intrasellar tumors >10 mm (5/7). All treatment-naïve patients had GH levels ≥2.5 µg/L (OGTT) or ≥5 µg/L (random/series), and IGF-1 levels >2 × ULN. There was no consistent pattern in the number of comorbidities in treatment-naïve patients.

#### Telephone interview (UK and Brazil only)

Endocrinologists found the instrument to be straightforward and easy to complete, with no major challenges. The scoring was easy to perform and report, but endocrinologists reported that management decision rules based on scores were needed. Feedback on the sections of the SAGIT instrument are summarized in Supplemental Table 2.

#### PRAC-Test^©^

Responses on the utility of the SAGIT instrument are summarized in Fig. 2 and country-specific data are shown in Supplemental Table 3. Most endocrinologists reported that the instrument would be useful for scientific purposes (10/13), to assess the response to treatment (8/13), and to contribute to therapeutic decision-making (8/13). None of the participants felt that the instrument would be of no use.

Responses on practical aspects of using SAGIT are shown in Fig. 3 (and for the individual countries in Supplemental Table 4). Most endocrinologists indicated that the instrument was concise (12/13), unbiased (12/13), simple (11/13), easy to understand (11/13), quick to complete (11/13), and informative (10/13), and many considered it precise (9/13) and practical (8/12), although less than half considered it exhaustive (6/13). Very few elements of the instrument were selected as requiring improvement; exceptions were the response choices, score and decision rules, and interpretation and recommendations. Some participants reported that the instrument required further improvements; the numbers indicating that *no* further improvement was needed for each section were as follows: title, 10/13; instructions, 7/13; questions, 5/13; scores and decision rules, 5/13; interpretation and recommendations, 5/13; and response choices, 3/13.

All endocrinologists reported that some parties would benefit from using the SAGIT instrument, including the scientific research community (10/13) and healthcare professionals (8/13). Five participants indicated that patients could benefit, and one that the pharmaceutical industry could benefit.

The following were given as reasons for not using the SAGIT instrument: impractical (5/13), lack of relevance for patients (2/13), lack of scientific credibility (2/13), and lack of clinical relevance (2/13). Six endocrinologists indicated that there was no reason not to use the instrument. In terms of their willingness to use the instrument, one participant indicated that he/she would not use it; the remainder would use it with most patients (n = 6/13), a minority of patients (n = 5/13), or all patients (n = 1/13). Furthermore, 10 of the 13 endocrinologists would recommend the instrument to their colleagues and 9/13 believed that they would have a positive reaction to it.

Participants felt that it would be most appropriate to complete the SAGIT instrument during a patient consultation (9/13), between consultations (4/13), or in the waiting room immediately before the consultation (1/13).

Following completion of the pilot study, the SAGIT instrument was revised in response to feedback. Revisions included a simplified scoring system for signs and symptoms (S) and associated comorbidities (A) and greater emphasis on the need to report GH after OGTT *or* as random/series measurement. The updated version of the instrument to be used in the clinical validation study is shown in Fig. 1b.

## Discussion

SAGIT is a new ClinRO instrument, that is, it allows “assessment of the status of a patient’s health condition based on clinician observation and interpretation” [23]. It was developed by acromegaly experts and structured to reflect key components associated with diagnosis and the management of patients with acromegaly, namely signs and symptoms (S), associated comorbidities (A), GH levels (G), IGF-1 levels (I), and tumor profile (T). This original version of the SAGIT instrument was field-tested, in line with published methodology [21], with practising endocrinologists managing patients with acromegaly using an iterative, robust, and rigorous process consisting of pre-testing and a subsequent pilot study. These two steps allowed the content and layout of the instrument to be tested in different settings, that is, retrospectively using patients’ medical records and prospectively during a consultation, at different timepoints (before and after an intervention, and in real-time) and with different patient profiles (stable/controlled, active/uncontrolled, and treatment-naïve).

Assessment of quality of life was not included in the SAGIT instrument as this is best rated by patients themselves and therefore less suitable for a ClinRO instrument. Furthermore, a patient-reported outcome instrument is already available for assessing quality of life in acromegaly (the AcroQoL questionnaire). To provide endocrinologists with a comprehensive perspective of acromegaly status and evolution, complementing the SAGIT instrument with a PRO questionnaire would be recommended as this may add an important dimension related to patient status. This is particularly important in view of the frequent mismatch between outcomes when reported by clinicians and patients, an issue raised by some endocrinologists during the cognitive debriefing in Step 1.

In the Step 1 pre-testing, the endocrinologists’ opinion of the SAGIT instrument in its original version was uniformly favorable. In particular, participants acknowledged the brevity, simplicity, rapidity, ease of use, objectivity, and one-page format of the instrument, features essential to endocrinologists if the instrument is to be used for patient management in clinical practice. These results are encouraging for an early-stage version of an outcome instrument, particularly as scoring and interpretive rules have yet to be developed. However, the pre-testing step also identified general issues (e.g. how to report the score for each section), as well as section-specific issues, such as the utility of the severity ranking for signs and symptoms and associated comorbidities. Methods of assessing GH (i.e. either nadir following OGTT or random/series) also required modification and clarification because of differences in practice across countries and according to patient profiles.

Results of the pilot study, which was conducted mainly in real-time, were encouraging, confirming the appropriateness of the instrument for clinical practice in terms of content, length, and rapidity of completion, and acceptability to endocrinologists. In particular, good consistency between pre-determined stage, GH and IGF-1, and signs and symptoms was reported. Interestingly, however, data on tumor dimensions and comorbidities were less predictable based on GH and IGF-1 and on pre-selected stage of the disease. This reinforces the concept that a comprehensive instrument may capture clinical features pertinent to therapeutic decision-making that cannot be easily predicted based solely on biochemical evaluation/staging. According to the endocrinologists who participated in Step 2, aspects of the instrument requiring improvement were decision and interpretation rules, and recommendations for patient management. The large-scale validation study (Step 3 clinical validation phase) will provide information necessary to develop this guidance. As well as confirming the practicality and utility of the instrument, the pilot study provided preliminary evidence that the SAGIT instrument differentiates between different profiles of patients with acromegaly (stable/controlled disease, active/uncontrolled disease, and treatment naive); this will also be explored further in Step 3.

The relatively small numbers of participating endocrinologists limits comparisons between results obtained in different countries. However, it is interesting that despite cross-country differences in management and practice, endocrinologists’ opinion and feedback was generally similar across countries. The only exception to this was observed in Step 1 pre-testing: two endocrinologists (from Spain) had the most negative views of the instrument, and the only participant who indicated that they would not be willing to use the instrument in any patient was from Spain. The link between disease severity and SAGIT scores was questioned by one of these endocrinologists during the interview, and may explain the scepticism. These findings were not replicated in Step 2, and the use of the instrument in real-time conditions on a larger scale (in the Step 3 clinical validation study) should test the results of the pilot study.

Recently, ACRODAT (ACROmegaly Disease Activity Tool) [15], a multidimensional instrument based on clinical features but not GH levels, has been proposed. However, information is limited and has not been published.

In the next phase of SAGIT, which will also be conducted retrospectively using medical records as well as prospectively in real-time, rules and recommendations for treatment decision-making and patient management will be defined. The ability of the instrument to discriminate groups of patients with acromegaly (controlled versus not controlled) and to define a new acromegaly staging based on clinical, biochemical, and tumor parameters in the instrument will also be assessed. The study will be conducted in 35 centers in 10 countries worldwide and enrolment of ≥200 patients is planned.

On the basis of the qualitative pilot testing phase, the current content and format of the SAGIT instrument are well accepted and understood by endocrinologists. SAGIT is a promising instrument offering the potential for standardized classification and management of acromegaly, thereby facilitating optimal patient management.

## Electronic supplementary material

Supplementary material 1 (PDF 197 kb)
