# Supplementary material for: SAGIT®: clinician-reported outcome instrument for managing acromegaly in clinical practice—development and results from a pilot study
Source: Pituitary. 2015 Sep 16;19:39–49. doi: 10.1007/s11102-015-0681-2 (PMC4710645; doi:10.1007/s11102-015-0681-2)
Supplement: Supplementary file 1 — Supplementary material 1 (PDF 197 kb) [file 11102_2015_681_MOESM1_ESM.pdf]

Supplemental figures and tables

Supplemental Fig. 1 Stepwise development process for the SAGIT instrument

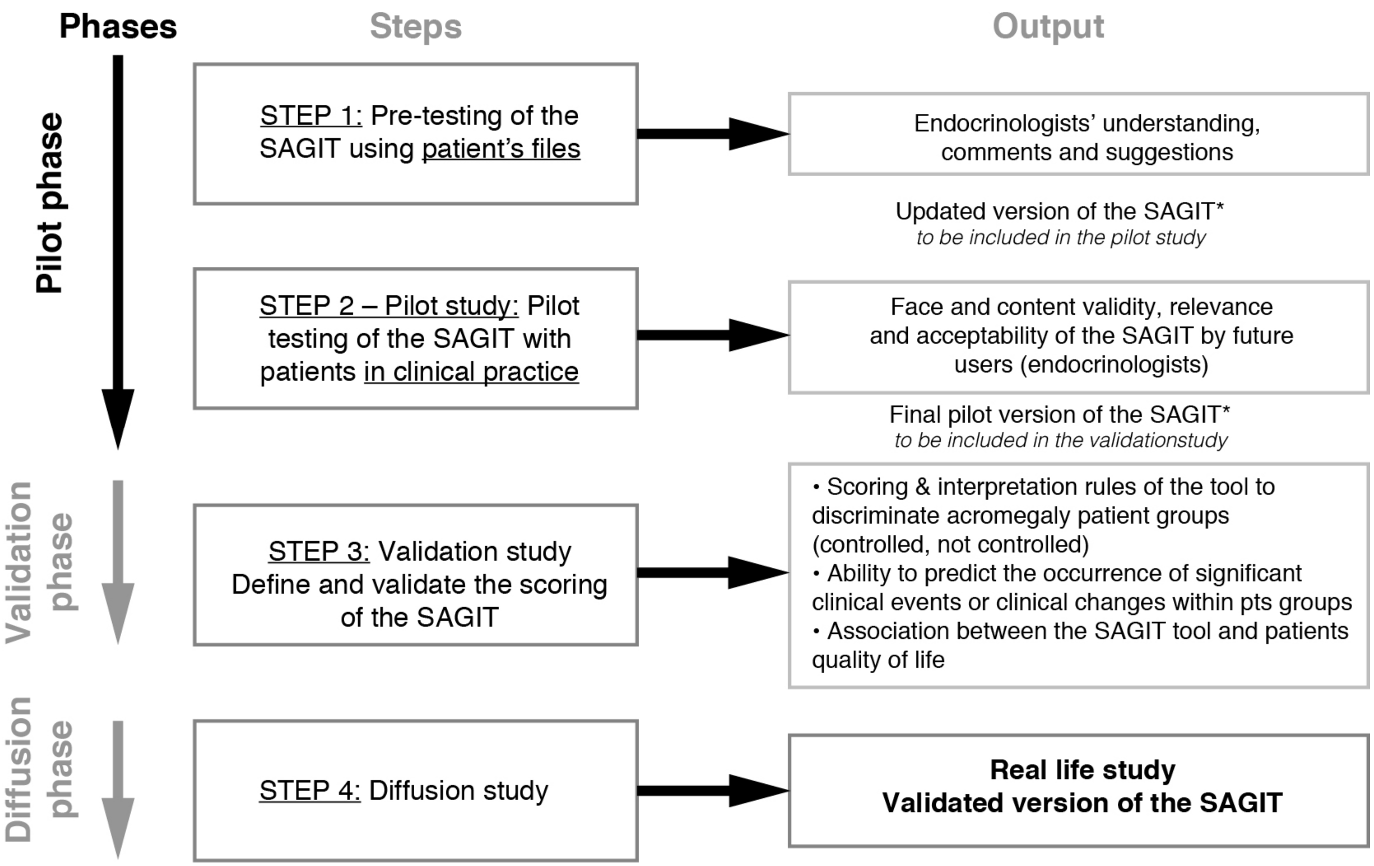

Supplemental Table 1 Characteristics of endocrinologist population included in Step 1 pre-testing and Step 2 pilot study according to country

	Step-1 pre-testing (n=11)						Step 2 prospective pilot study (n=9)					Step 2 retrospective pilot study (n=4)	
Characteristics	Brazil	France	Germany	Italy	Spain	UK	France	Germany	Italy	Spain	USA	Brazil	UK
Number of endocrinologists	2	2	1	2	2	2	2	1	2	2	2	2	2
Age (years)[a]	47–48	40–58	32	40–48	53	45–47	41–54	43	34–44	34–56	42–44	38–48	48[b]
Mode of practice[c]													
Outpatient clinic	1	0	0	0	1	2	0	1	1	1	0	2	2
Hospital	1	2	1	2	1	2	2	1	2	1	2	0	0
Number of years treating acromegaly patients[a]	15–20	10–30	2	8–20	23–25	11–18	12–24	12	7–10	3–30	3–30	10–14	10–19
Number of acromegaly patients seen per month[a]	10–60	2–30	5–10	10–40	10–12	2–5	3–10	10	3–6	4–10	10–15	12–80	1–10

[a]Range; [b]Missing data; [c]Some endocrinologists worked in both outpatient clinics and hospitals

Supplemental Table 2 Endocrinologists' feedback (via telephone interview) on items in each section of the SAGIT instrument during Step 1 pre-testing and Step 2 retrospective pilot study

	General comments/difficulties reported	
**Sections of SAGIT**	**Step 1 pre-testing (original instrument)**	**Step 2 retrospective pilot study (pilot instrument)**
**Title**	• Informative<br>• No need for improvement	• No issues reported, except for the need to adapt the first page for clinical study
**Signs and symptoms (S)**	• Lack of instructions on how to score each item, how to interpret the score, and how to account for the severity ranking<br>• List of symptoms fine; some additional symptoms proposed (e.g. acral changes, fatigue/asthenia, visual symptoms, paraesthesia in feet or legs, skin changes, facial dysmorphy, cardiopathy)	• No major issues<br>• Need for "cosmetic" changes only<br>• Comorbidity "swelling" needs to be more explicit or reworded because it does not apply exactly to what patients with acromegaly have
**Associated comorbidities (A)**	• Lack of instructions on how to score each item, how to interpret the score, and how to account for the severity ranking<br>• List of comorbidities fine; additional comorbidities proposed (e.g. visual signs, intestinal polyps, obesity, cancer)<br>• Definition needed for each comorbidity listed	• Comorbidity "swelling" needs to be more explicit or reworded because it does not apply exactly to what patients with acromegaly have
**GH levels (G)**	• Well understood<br>• Ranges and units well adapted but loss of sensitivity for small improvements because ranges of the concentration categories are too large<br>• GH nadir with OGTT and GH random or series not always reported in the patient's medical record, or not always performed routinely at each consultation	• Need to highlight the "OR" between GH nadir with OGTT and GH random or series

**GH levels (G)**	• Highest concentration of GH nadir with OGTT proposed is not high enough<br>• Meaning of "series" not understood	
**IGF-1 levels (I)**	• Well understood<br>• Ranges and units well adapted, but loss of sensitivity for small improvements because concentration ranges are too large	• No changes required
**Tumor profile (T)**	• Some categories are not sensitive enough<br>• Important to differentiate invasive from non-invasive tumors, parasellar/laterosellar versus intrasellar versus suprasellar tumors, sinus versus chiasm versus cavernous sinus versus sphenoidal sinus invasion	• More precision is required

GH, growth hormone; IGF-1, insulin-like growth hormone-1; OGTT, oral glucose tolerance test

Supplemental Table 3 Utility of the SAGIT instrument: country-specific results of PRAC-Test® questionnaire during Step 1 pre-testing and Step 2 pilot study

	Step-1 pre-testing							Step 2 pilot study								
								Prospective						Retrospective		
SAGIT uses	Brazil (n=2)	France (n=2)	Germany (n=1)	Italy (n=2)	Spain (n=2)	UK (n=2)	TOTAL (n=11)	France (n=2)	Germany (n=1)	Italy (n=2)	Spain (n=2)	USA (n=2)	SUBTOTAL (n=9)	Brazil (n=2)	UK (n=2)	TOTAL (n=13)
To contribute to therapeutic decision-making	2	1	1	1	0	0	5	1	1	1	2	1	6	0	2	8
For scientific purposes	2	1	1	0	0	2	6	2	1	1	2	1	7	2	1	10
To assess response to therapy	1	2	1	2	2	1	9	1	0	2	1	1	5	2	1	8
As a document to be included in patient's medical record	1	2	1	1	0	2	7	1	0	0	1	1	3	2	0	5
To help healthcare professionals to adapt treatment	1	2	1	1	0	1	6	1	0	1	1	0	3	0	1	4
To contribute to the diagnosis process	2	1	1	1	0	0	5	0	0	2	0	1	3	0	0	3
To monitor compliance or adherence	0	0	0	1	0	2	3	0	0	1	0	0	1	0	1	2
To screen patients	1	0	0	0	0	1	2	0	0	0	0	1	1	0	0	1
To assess side effects	0	0	0	1	0	0	1	0	0	0	0	0	0	0	0	0
Of no use	0	0	0	0	0	0	0	0	0	0	0	0	0	0	0	0

Supplemental Table 4 Practical aspects of the SAGIT instrument: results of the PRAC-Test® questionnaire in each country during Step 1 pre-testing and Step 2 pilot study

Practical aspects of SAGIT	Step-1 pre-testing							Step 2 pilot study								
								Prospective						Retrospective		
	Brazil (n=2)	France (n=2)	Germany (n=1)	Italy (n=2)	Spain (n=2)	UK (n=2)	TOTAL (n=11)	France (n=2)	Germany (n=1)	Italy (n=2)	Spain (n=2)	USA (n=2)	SUBTOTAL (n=9)	Brazil (n=2)	UK (n=2)	TOTAL (n=13)
Easy to understand	2	2	1	1	1	2	9	2	1	2	2	1	8	1	2	11
Concise	2	2	1	2	2	2	11	2	1	2	1	2	8	2	2	12
Precise	1	2	1	1	1	1	7	1	1	2	1	1	6	1	2	9
Informative	2	2	1	2	1	2	10	2	0	2	2	1	7	1	2	10
Practical	2	1[a]	1	2	1	2	9[a]	0	0	2	2	1	5[a]	1	2	8[a]
Simple	2	1[a]	1	1	2	2	9[a]	2	0	2	2	1	7	2	2	11
Exhaustive	0	0	0	1	1	1	3[a]	1	1	2	0	1	5	0	1	6
Quick to complete	2	1[a]	1	2	2	2	10[a]	2	0	2	2	1	7	2	2	11
Unbiased	1	1[a]	1	2	2	2	9[a]	1	1	2	2	2	8	2	2	12

[a]One data point missing

Supplemental Table 5 Baseline characteristics of patients who took part in Step 2 pilot study

Characteristics	Disease/treatment status			Total (n=26)
	Stable/controlled acromegaly (n=10)	Active/uncontrolled acromegaly (n=9)	Treatment naïve (n=7)	
Age (years)				
Mean	57.8	45.0	45.3	50.0
Median (range)	58.5 (44–73)	44.0 (19–77)	44.0 (31–63)	49.0 (19–77)
Gender (n)				
Male	5	4	3	12
Female	5	5	4	14
Time since acromegaly diagnosis (years)				
Mean	7.5	2.6	0.1	3.8
Median (range)	6.0 (1–22)	2.0 (1–5)	0.0 (0–1)	2.0 (0–22)
